# What is the coverage of your health insurance plan? An audit of hospital billing

**DOI:** 10.3934/publichealth.2024052

**Published:** 2024-09-24

**Authors:** Aswin Sugunan, Rajasekharan Pillai K, Anice George

**Affiliations:** 1 School of Management, The Apollo University, Chittoor, India; 2 Manipal Academy of Higher Education, Manipal, India; 3 Welcomgroup Graduate School of Hotel Administration, Manipal Academy of Higher Education, Manipal, India; 4 Manipal College of Nursing, Manipal Academy of Higher Education, Manipal, India

**Keywords:** private health insurance, out-of-pocket expenditure, underinsurance, health insurance coverage, hospital billing data

## Abstract

The provocative advice of health policymakers in endorsing private health insurance, as a critical tool for health reforms, is well-reckoned as a deterrent to mounting healthcare expenditure in the wake of the public health insurance quagmire. However, scholarly evidence has condemned the ineffectiveness of private health insurance in containing out-of-pocket expenditure. In this backdrop, we carried out a nuanced investigation of the coverage pattern of private health insurance policies. We examined the one-year billing information of private health insurance holders hospitalized in a multi-specialty teaching hospital. We found that private health insurance fails to provide full coverage, leading to underinsurance though minimal financial protection was extended. Moreover, reimbursement patterns under various cost heads are also discussed. We conclude by emphasizing the need for future research to fill the knowledge gap. We claim methodological novelty in its approach to data collection.

## 1. Introduction

Individuals seeking healthcare, particularly those with limited financial resources, often suffer significant financial strain due to out-of-pocket expenses (OOPE) [1],[2]. Shockingly, an estimated 17 percent of the global population experiences catastrophic health expenditure (CHE), with a vast majority of cases originating in Asia and Africa [3]. The combination of OOPE, poor health, and inadequate government spending has led health policymakers to tout health insurance as a critical tool for reform in developing countries [4]. As a result, numerous government-supported and community-based health insurance schemes (CBHI) have emerged, designed to provide financial protection and mitigate the burden of OOPE [4]. However, the effectiveness of these insurance programs remains uncertain, with some studies indicating a reduction in OOPE [5], while others report their failure [6]–[8]. Within the realm of healthcare policy, there is a push among policymakers to incorporate private health insurance as a complementary option to public-funded health insurance (PFHI) to improve overall health coverage for the populace [9],[10]. However, it remains unclear whether private health insurance can effectively alleviate the burden of healthcare expenses. To shed light on this issue, our current study delves into the intricacies of private health insurance coverage by closely analyzing hospital bills in relation to both patient and insurance claims.

Scholars have investigated this knowledge domain from multiple perspectives, such as the penetration and acceptance levels of private health insurance [11],[12], factors influencing the choice of health insurance [11],[12], the role of private health insurance in healthcare utilization [13],[14], and OOPE [9],[13],[15],[16]. Although the performance of health insurance in enabling healthcare utilization and containing cost is a trending topic among the research community, most of the studies are skewed towards not-for-profit (public) health insurance, leaving a knowledge vacuum in the private health insurance landscape. A few researchers have attempted to bridge this gap by evaluating private health insurance [9],[13]–[16]. Healthcare burden due to forgone care and less healthcare utilization due to higher insurance cost sharing is reflected among US citizens [14]. In addition, OOPE is reported to be six times higher in Australian and Chinese citizens with private health insurance than PFHI [15],[16]. However, Grigorakis [17] and Volker [9] have endorsed private health insurance as a complement to PFHI in improving health coverage. This is successful in Greece, Vietnam, and Israel in tackling the stifling OOPE [9],[17].

Despite these significant contributions to the outcomes of private health insurance, methodological concerns persist due to the limitation of data collection techniques used in large-scale surveys. Scholars have challenged the credibility of primary data obtained through surveys owing to its inherent recall bias [9],[13]–[16]. Roa [18], Sinha [19], and Wagstaff [20] also have questioned this obscurity in information extracted from large-scale national surveys. This demands a fresh insight into the role of private health insurance in allaying healthcare costs by evaluating data available at the institutional level. The current study is an earnest endeavor in this regard. To accomplish the overarching goal of the study, we seek answers to the following research questions:

• What is the share of out-of-pocket disbursal in total inpatient expenditure?

• What is the pattern of coverage as per various cost heads?

• Is there any association between patient profile and healthcare expenditures?

Our study makes a few contributions. First, to the best of our knowledge, this is the first study in the field of private health insurance based on a hospital database to address an observed methodological and theoretical void. Second, this study provides insight into the underinsurance phenomenon latent in private health insurance and coverage patterns under different cost heads. These insights may help health policymakers design optimized and affordable health insurance plans for patients. Thirdly, from the patients' perspective, we provide an overview of the reimbursement pattern by private health insurance, suggesting ideal healthcare plan options to consider.

### 1.1. Health insurance in India

The Indian insurance industry is broadly classified into life insurance and non–life insurance or General Insurance, which includes Health, Marine, Motor, and Fire insurance. Government-sponsored health insurance in India began in 1948 with the introduction of Employee State Insurance (ESI) and, subsequently, the Central Government Health Scheme (CGHS) in 1954. After nationalizing the general insurance industry, the Mediclaim policy was introduced by the General Insurance Company in 1986 [21]. Entry of private insurance happened soon after the liberalization of industry in 2000 by the Insurance Regulatory Development Authority (IRDA). Presently, there are six standalone private health insurance companies in the country [22]. Health insurance plans in India include hospitalization policy, hospital daily cash benefit policy, critical illness policy, and surgical cash benefit policy [22]. Hospitalization policy consists of family floater health insurance and individual health insurance. Due to many health insurance products and a variety of features, IRDA launched a standard health insurance scheme called “Arogya Sanjeevani” to have a common policy wording across the industry and provide financial assistance to the public [21],[22].

Financial risk protection against household healthcare expenditure has become the core focus of all elements of UHC across the globe, drawing the researcher's ample attention to health insurance [23],[24]. Many studies have tried to assess the outcome of health insurance schemes [25]–[27], but they confined their focus to PFHI such as Rashtriya Swasthya Bima Yojana (RSBY), Vajpayee Arogyashree (VAS), Rajiv Arogyashree (RAS), and PMJAY. These studies have contended that such insurance schemes could not accomplish the primary objective of cashless treatment [25]–[29]. Hence, scholars have advocated expanding private health insurance to improve health coverage [30]. Thus, it is imperative to explore the pros and cons of private health insurance.

### 1.2. Literature review

A large growing body of literature has investigated health insurance in India from both economic and social points of view. These include the evaluation of PFHI [31]–[33], community-based health insurance [34],[35], micro-insurance [36], and private health insurance [13] in OOPE prevention and healthcare utilization [13],[14], satisfaction of policy holders regarding the claim settlement across public and private health insurance [37], willingness of health insurance purchase [11],[38], challenges of health insurance in India [39], and coverage trends of private health insurance [40]. Studies on financial protection from health care expenditure by PFHI offer no conclusive evidence to declare the policies that had any impact on preventing OOPE [31]–[33]. The outcome on the role of CBHI in preventing OOPE received mixed outcomes. When Devadasan [34] argues that CBHI significantly reduces the healthcare burden, Eze [35] contradicts the findings in the latest review. We identify that CBHI is least effective in protecting healthcare seekers from OOPE across lower-middle-income countries, including India [35]. A similar trend is observed in the process of micro health insurance [36] and private health insurance [13] to provide financial protection to its policyholders.

A few researchers have explored policyholders' satisfaction [37] and their willingness to purchase public and private health insurance schemes [11],[38],[40]. The results reveal that most insurance holders are more satisfied with the public health insurance sector than the private. Dror [38] argues that the willingness to purchase health insurance is perceptibly higher among those who have already been insured than the uninsured or never insured. This is because the insured are more aware of the likely benefit of health insurance than the uninsured. The work of Yadav [11] offers additional insight into the likelihood of a policy renewal rather than a fresh subscription by highlighting the awareness of the need for health insurance, the risk coverage, and the tax benefits provided by the policy. In addition, the number of members in the family, health status, financial status, and age also have a significant relationship to the purchase decision [41],[42]. Binny [39] has explored the challenges faced by private health insurance companies in expanding coverage and identified a few hurdles, such as a high incurred claim ratio, the rising cost of insurance products, the lack of facilities to understand the disease pattern among the population and changing needs of customers [39]. Another study on the coverage pattern of health insurance has indicated the burden of outpatient costs, which is not a full-fledged offering under private health insurance [40]. Hence, the literature offers no evidence on the role of even PFHI in ensuring financial protection.

The ineffectiveness of PFHI, as a financial safeguard has resulted in encouraging private health insurance to act as a complement to improve coverage and reduce OOPE by health policymakers [41]. Considering the growing importance of private health insurance and the lack of evidence on whether it reduces financial distress, it is necessary to investigate the role of private health insurance in containing excess medical expenditure. Therefore, we examine the role of private health insurance on health expenditure using institutional-level billing data.

## 2. Methodology

### 2.1. Research design and settings

We employed a cross-sectional research design using one-year billing information of hospitalized patients with private health insurance policies. The necessary data were extracted from the billing database and Electronic Medical Record (EMR), for 12 months, April 2022 through March 2023, of a 2000-plus-bed multi-specialty tertiary care teaching hospital located in the coastal region of south Karnataka, India. The hospital caters to more than six lakh patients annually and is considered a major network hospital providing service for leading health insurance companies in India. During the study phase, it is understood that the hospital undertakes services for more than 15 health insurance companies.

### 2.2. Data source

To answer the research questions formulated for this investigation, we examined the data on billing and insurance claims of 13,115 private health insurance holders hospitalized in a multispecialty tertiary teaching care hospital for one year. The data comprises the proportion of health insurance coverage under various cost heads such as consumables, services, medicine, and bed costs. The expenditure data were extracted at two levels:

Level 1: Total inpatient expenditure (TIE): The inpatient billing department extracted the costs incurred for patients per hospitalization. This data comprised information on the total expenditure incurred for inpatient services, the reimbursed amount received from the health insurance company, and the remaining amount paid by the patient.

Level 2: TIE breakups: TIE is the summation of the amount billed against the hospitalized patient under various sub-categories. These include costs incurred for inpatient services, care packages, costs incurred for inpatient medicines and materials used, and costs incurred for patient accommodation (bed costs). To investigate the share of each sub-category in TIE and reimbursement received, we accessed the primary billing databases using the unique identification number.

#### 2.2.1. Definition of terms

1. Total Inpatient Expenditure (TIE): TIE is the total amount incurred for inpatient services. This includes the cost incurred for treatment packages, services, materials, medicines, and beds.

• Package cost: The amount incurred for a bundle of services for a specific disease. For example, our data shows that treatment packages are common in cardiac conditions and procedures such as angiograms or angioplasty.

• Service cost: It includes the amount incurred for services such as admission charges, consultation fees, documentation charges, nursing charges, diet services, inpatient diagnosis charges, inpatient lab costs, etc.

• Material cost: Includes the amount incurred for consumables for inpatient services such as gloves, masks, pads, cardiac stents, etc.

• Medicine cost: Is defined as the amount incurred for the purchase of medicine during hospitalization on an inpatient basis but excludes any medicines purchased on an outpatient basis.

• Bed cost: It includes the cost incurred by a patient during hospitalization. The bed facilities are categorized into ICU bed, general, semi-private, semi-special, semi-deluxe, special, super-deluxe, deluxe, and luxury suite.

2. Coverage amount: Is reimbursed by the insurance company based on the health plan against the cost heads.

3. OOPE: Includes the amount paid by the patient as an in-patient after the reimbursement received from the health insurance company.

### 2.3. Sampling method

The primary data extracted from the inpatient billing department comprises the relevant information on 13,115 patients. However, the primary billing data (N = 13,115) consists only of the total cost incurred in inpatient services and the total amount reimbursed. Unless categorized, these data cannot confer useful information on the pattern of coverage as per various cost heads (Research question 2). However, cataloguing the entire data (N = 13,115) appeared puzzling and laborious. Therefore, to study the reimbursement pattern of health insurance plans under different cost heads, we selected 5% of patients from each disease category through proportionate simple random sampling (n = 656). This breakdown is presented in Table 1.

**Table 1.** A proportionate sample number of patients.

Sl. No	Disease conditions	Total Cases	(5%)
1.	Cardiac conditions	1497	75
2.	Disease for internal organs	4468	224
3.	Cancer conditions	1727	86
4.	Renal conditions	1418	71
5.	Musculoskeletal conditions	1418	71
6.	Obstetrics and gynecology	2054	102
7.	Neurological conditions	360	18
8.	Psychiatric conditions	156	7
	Total	13115	656

Source: Present study.

### 2.4. Data analysis

The data analysis comprises two sets of data: population data and sample data. Population data (N = 13,115) are used to elicit an overall picture of this study's focus and estimate TIE and the share of OOPE in TIE. The sample data (n = 656) examines the disease-specific reimbursement pattern, the relative weightage of a specific disease, and cost heads in mustering OOPE. The data were coded and analyzed using Jamovi (V. 2.4.14). We used descriptive statistics to estimate the median and mean expenditure incurred by the patient. The patient profile was expressed using frequency distribution. The normality of the variables was investigated using the Shapiro-Wilk test, which highlighted the violation of normality. The association between the patient profile indicators and health expenditure was assessed using Spearman rank correlations for ordinal variables and Kendal Tau for nominal variables.

## 3. Results

We present the results of data analysis with the guiding research questions in mind, as presented under the ensuing three subheads.

### 3.1. Patient profile and its association with healthcare expenditure (n = 656)

Here, we present the patient profile for 656 samples. The majority (46.5 percent) of the patients fall within the age group of 51 to 65 years of age. The superiority of males is observed in the selected samples, representing 65.1 percent. Information regarding the length of stay (LOS) indicates that a major share of patients reported having LOS less than three days. The details of the patient profile are presented in Table 2.

**Table 2.** Profile of patient sample selected for detailed investigation (n = 656).

Patient Profile Age (in years)	Counts	Percentage Total	Cumulative Percentage
18–35	47	7.2 %	7.2 %
36–50	110	16.8 %	24.0 %
51–65	304	46.5 %	70.5 %
66–80	180	27.5 %	98.0 %
>80	13	2.0 %	100.0 %
Gender			
Female	228	34.9 %	34.9 %
Male	426	65.1 %	100.0 %
Length of stay (in days)
0–3	260	39.8 %	39.8 %
4–6	210	32.1 %	71.9 %
7–9	74	11.3 %	83.2 %
>9	110	16.8 %	100.0 %

Source: Present study.

### 3.2. Out-of-pocket share in TIE

Here, we highlight the indicators of the financial burden of patients with private health insurance admitted for one year (April 2022 through March 2023). Out of 13,115 data points analyzed, 98.5 percent (N = 12,913) experience OOPE for in-patient services despite being insured. The magnitude of OOPE is estimated by assessing the proportion to TIE at an interval of 10 percent (Table 3). It is observed that 50 percent of the inpatients experience an OOPE up to 30 percent of TIE. The median OOPE faced by the inpatient is ₹5773 (₹1 = USD 0.012), and the mean OOPE is ₹17,558.78 (σ = ₹41,979). The median and mean reimbursement from insurance companies is ₹18,323 and ₹40,574.69 (σ = ₹60,058), respectively.

**Table 3.** Share of OOPE to TIE among the private insurance holders.

Sl. No	Patient's share to TIE (%)	Frequency	Percentage Frequency
1.	0–10	2785	21.24
2.	10–20	2676	20.40
3.	20–30	2237	17.06
4.	30–40	1590	12.12
5.	40–50	1296	9.88
6.	50–60	889	6.78
7.	60–70	516	3.93
8.	70–80	572	4.36
9.	80–90	449	3.42
10.	90–100	105	0.80
	Total	13,115	100

Source: Present study.

The median and mean estimates of inpatient healthcare cost (TIE), OOPE, and coverage amount reimbursed for in-patients with various disease conditions are presented in Table 4.

**Table 4.** Disease-wise inpatient healthcare cost (amount in ₹ thousands).

Disease conditions	Numerical summaries	TIE	OOPE	Coverage amount
Cardiac	Min	14.322	0	0
	Median (Mean)	145.468 (160.374)	7.598 (25.285)	137.870 (135.089)
	Max	1002.245	463.843	867.245
	SD	159.952	58.495	135.742
Disease for internal organs	Min	4.494	0	3.484
	Median (Mean)	63.192 (101.154)	8.925 (23.897)	54.267 (77.257)
	Max	1121.015	896.015	528.541
	SD	128.880	67.565	86.114
Cancer	Min	3.630	0	2.700
	Median (Mean)	41.631 (69.415)	3.410 (12.824)	38.221 (56.591)
	Max	457.499	200.264	417.760
	SD	80.784	29.000	70.557
Renal	Min	6.354	0	5.263
	Median (Mean)	55.045 (68.151)	7.195 (13.890)	47.850 (54.261)
	Max	445.893	131.721	314.172
	SD	67.155	19.402	51.250
Reproductive	Min	5.293	0	3.387
	Median (Mean)	56.902 (63.571)	8.305 (14.688)	48.597 (48.883)
	Max	222.828	98.895	207.355
	SD	42.227	17.709	34.287
Musculoskeletal	Min	8.369	0	5.076
	Median (Mean)	117.027 (130.348)	12.021 (26.506)	105.006 (10.841)
	Max	508.871	397.821	256.044
	SD	92.681	59.609	62.384
Neurological	Min	15.559	1.000	0
	Median (Mean)	66.450 (95.316)	5.811 (14.568)	60.639 (80.747)
	Max	382.818	70.391	312.427
	SD	88.995	18.624	75.292
Psychiatric	Min	10.403	1.198	9.205
	Median (Mean)	31.336 (30.065)	5.188 (8.710)	26.148 (21.355)
	Max	44.983	34.983	35.267
	SD	11.806	11.778	9.886

Source: Present study.

Patients with cardiac disease conditions incurred the highest median TIE (₹145,468). This is followed by patients with musculoskeletal disease conditions (₹117,027) and neurological conditions (₹66,450). Out-of-pocket share among the TIE is dominated by patients with ailments related to musculoskeletal (₹12,021), followed by patients with general disease conditions (₹8925) and reproductive health with a median cost of ₹8305. The insurance company covers a substantial amount of the TIE. For example, the median reimbursement cost received by cardiac patients is ₹137,870. Patients hospitalized with musculoskeletal conditions received a median reimbursement of ₹105,006. Similarly, patients with other disease conditions related to reproductive health and general ailments received ₹48,597 and ₹54,267, respectively. The private health insurance company provides significant coverage for the expenditure incurred by an inpatient insurer.

### 3.3. The pattern of health insurance coverage in TIE

The TIE is categorized into costs incurred for packages, services, materials, medicine, and beds. Therefore, we analyzed the coverage provided by health insurance in each category (Table 5).

**Table 5.** Share of health insurance coverage in inpatient cost (n = 656; amount in ₹ thousands).

Items	Numerical summaries	Total amount	Coverage amount	OOPE
Packages	Min	0	0	0
	Median (Mean)	0 (6.836)	0 (6.760)	0 (0.076)
	Max	400.000	400.000	16.000
	SD	29.309	29,.019	0.972
Services	Min	0.560	0	0
	Median (Mean)	31.905 (47.532)	29.915 (43.128)	1.990 (4.404)
	Max	54.123	375.447	426.237
	SD	58.260	46.582	28.276
Materials	Min	0	0	0
	Median (Mean)	4.931 (7.731)	2.655 (5.433)	2.276 (2.298)
	Max	104.828	82.902	104.828
	SD	11.664	8.981	7.088
Drugs	Min	0	0	0
	Median (Mean)	13.430 (26.228)	7.673 (17.385)	5.757 (8.843)
	Max	421.316	261.516	421.316
	SD	42.207	30.454	25.705
Bed	Min	0	0	0
	Median (Mean)	4.600 (8.710)	0 (3.131)	4.600 (5.579)
	Max	163.800	60.105	163.800
	SD	12.731	7.209	10.502
Total Inpatient Expenditure	Min	33.630	0	0
	Median (Mean)	62.959 (96.575)	50.000 (76.633)	12.959 (19.942)
	Max	1121.015	867.245	896.015
	SD	111.012	83.890	50.716

Source: Present study.

The median expenditure incurred for the packages across cardiac patients is zero. However, the average cost is reported to be ₹6836. Health insurance has covered around 98 percent (mean = ₹6760) of the cost incurred for the treatment packages. For services, patients incurred a median expenditure of ₹31,905, out of which health insurance coverage stands at a median cost of ₹29,915. A similar trend is observed for the cost incurred for materials, drugs, and patient bed charges. Total median costs incurred for drugs purchased by inpatients are reported to be ₹13,430, 2.7 times the median cost incurred for materials (₹4931) and 2.9 times for beds (₹4600). The patient median bed cost is notably higher than other categories (₹4600).

### 3.4. Relationship between key patient profile and health expenditure and coverage

We examined the relationship between key patient characteristics and coverage amount, THC and OOPE. Spearman rank order correlation [43] was used for LOS and age characteristics, whereas Kendall's Tau [43] was used for disease types and gender. The results are presented in Table 6.

**Table 6.** Association between patient profile and healthcare expenditures.

Indicators	Coverage amount (ρ)	TIE (ρ)	OOPE (ρ)
Length of stay	0.266 (<0.001)	0.202 (<0.001)	0.139 (<0.001)
Age	0.011 (0.789)	0.021 (0.583)	−0.036 (0.355)
Disease	−0.137 (<0.001)	−0.061 (<0.078)	0.034 (0.321)
Gender	0.033 (0.401)	−0.002 (0.958)	−0.009 (0.824)

Source: Present study.

LOS is a pertinent patient characteristic, which is subjected to varies with disease pattern, comorbidities and age characteristics. The relationships between LOS and the coverage amount (r = 0.266; ρ < 0.001), TIE (r = 0.202; ρ < 0.001) and OOPE (r = 0.139; ρ < 0.001) turn out to be statistically significant. It is also understood that these correlation coefficients indicate negligible correlations between the variables of reference. The relationship between age and other cost variables such as coverage amount, total hospital costs and OOPE is found to be statistically insignificant. Similarly, a significant relationship is highlighted between type of diseases and coverage amount (r = −0.137; ρ = < 0.001). The analysis also reflected a statistically non-significant association between gender and coverage amount (ρ = 0.401), TIE (ρ = 0.958) and OOPE (ρ = 0.824).

## 4. Discussion

We provide crucial information regarding the role of private health insurance in healthcare costs. The existence of minimal protection of health expenditure highlights the overt prevalence of OOPE among private health insurance holders. This is due to the partial coverage, excluding the cost incurred for inpatient services. Moreover, it is observed that the reimbursement towards bed costs in the TIE, according to the policies, is 30 percent. In addition, most patients received only 70 percent of the TIE through reimbursement. Upon examining the relative inpatient expenditure incurred for disease conditions, we identify the sway of cardiac disease in TIE.

One of the major findings in this research is the unraveling of the underinsurance of hospitalized patients. This questions the awareness and knowledge of health insurance holders while purchasing an optimum policy scheme, on the one hand, and its mis-selling of suboptimal policies on the other. Our results concur with Prinja [6], Erlanga [44], and Sommers [45] regarding the incidence of underinsurance among PFHI holders and with Vellakkeel [13] regarding private health insurance. The major causes of underinsurance among private health insurance holders, reported by Adrion [46] and Chhabra [14], include the highest cost-sharing (premiums, coinsurance, and co-payments) and unexpected outpatient costs. Though we could not extract the cost-sharing perspective of health insurance plans, the impact of partial reimbursement, in aggravating the severity of OOPE, is depicted in our study.

Our findings on the underinsurance phenomenon direct our attention to a few studies on the consequences of underinsurance sans space-time [14],[44],[46],[47]. Link [47] has reported the prevalence of co-morbidities and depression due to underinsurance among lower wealth quintiles and Hispanic blacks across the United States. Robertson [48] highlighted the possibility of overconsumption of healthcare and wastage, triggering under-insurance among health insurance holders.

Our study offers empirical evidence to the foregoing observations; the hike in bed costs is due to upgradation with an assumption of full health coverage and prolonged stay in intensive care units. There may be a lack of consumer health insurance literacy prior to purchasing a healthcare package, as indicated by the outcomes. Researchers have suggested that improving health insurance literacy could optimize healthcare utilization and avoid unnecessary healthcare-seeking costs [49].

We have identified a considerable share of bed costs for OOPE among healthcare seekers. Scholars have communicated similar findings by exposing the superiority of non-medical costs in the economic burden of diseases [50],[51]. We have noticed that prolonged ICU stays and partial coverage of bed costs by health insurance schemes have shot up healthcare costs. The partial coverage of bed cost is due to the upgradation of inpatient bed facilities from the fully covered basic level inpatient room (general ward) to deluxe or super deluxe rooms by patients. Reddy [52] has discussed similar observations in different settings.

Assessing the cost trends in healthcare seeking across the disease conditions reflects a higher burden of inpatient expenditure among patients hospitalized with cardiac diseases (median = ₹145,468). Our study fortifies the findings of past studies on cardiac patients' financial burden, which notify that the hike in inpatient expenditure accounted for higher costs incurred for human resources and capital expenses of hospitals [6],[53]. We observed that the patients seek admission to general wards and later get shifted to private rooms. This leads to excess inpatient expenditure incurred due to the upgrade of inpatient bed facilities, which contributes to a certain percentage of the increase in the cost of all other services they availed. Hence, the overall treatment cost is escalated, which adds to their healthcare burden.

Our study has the following limitations. First, we have included inpatient healthcare expenditure incurred for a specific episode of illness. This cannot predict the total economic burden experienced by private health insurance holders. For example, a patient might have been admitted to the hospital multiple times yearly due to co-morbidity or medical complications. The current study covers only one hospitalization episode for one patient. Hence, we do not claim to have covered the comprehensive OOPE of a patient. Second, the reimbursement amount varies according to the sub-schemes the health insurance provides, which is not considered. However, the analysis highlights a macroscopic view of the coverage and coverage limitations of private health insurance under each cost category. Fourth, the data were extracted from a multispecialty tertiary teaching hospital, limiting the generalization of the findings.

In light of this study, we propose a few future research directions. We have mentioned elsewhere the effectiveness of combinations of multiple interventions in reducing OOPE [54],[55]. Hence, researchers can investigate the question: Can the combination of non-insurance methods and private health insurance eliminate OOPE?

Similarly, countries have identified the benefits of private health insurance as a supplementary means of increasing coverage among the population [56],[57]. In addition, countries such as India report OOPE among PFHI holders [6]. In this regard, researchers can seek answers to the following research questions: What are the possibilities of increasing private health insurance penetration among partially insured individuals across lower and middle-income countries? What are the strategies adopted for the co-existence of both the insurance sectors – Public and private?

Though momentum is observed in the private health insurance market, whether it improves the volume of healthcare utilization, accessibility to healthcare, and overall population health requires further research. Therefore, future studies can be initiated to answer the following questions: What is the role of private health insurance in propelling health-seeking behavior? How far has health insurance been able to attain the existing healthcare delivery issues such as equity, accessibility, and affordability? What is the role of private health insurance in accelerating the goal of Universal Health Coverage? Do private health insurance holders experience any regret about the choice of policy? What is the level of health insurance literacy among private health insurance holders? What are the strategies to improve health insurance literacy among the population? Do consumers have any validated sources to identify an optimum health plan? How can we confluence health insurance schemes under private health insurance? Why are the insurance companies hesitant to embark on a shared-value insurance model?

## 5. Conclusions

We investigated the role of private health insurance in providing coverage against healthcare costs. We used billing information of patients hospitalized with private health insurance in a tertiary care multi-speciality hospital for one year. We appraised the coverage pattern of private health insurance under various cost heads. We found that the health insurance fails to provide full coverage, leading to under-insurance, though minimal financial protection is present. We have also identified less coverage for bed costs, resulting in OOPE. Patients' difficulty in choosing an optimum health insurance covering the out-patient cost is also highlighted. As per the authors' knowledge, this is the first attempt to exhibit the role of private health insurance in patients' health care costs using a pool of billing and insurance reimbursement data from a service provider. This strengthens the credibility of our findings compared to the results obtained from large-scale national sample surveys and provides the existing trend of private health insurance's role in providing coverage. We conclude by discussing campaigning for a suitable policy regime to set out an appropriate shared-value insurance model to ameliorate the underinsurance issue and devastating OOPE.

## Use of AI tools declaration

The authors declare they have not used Artificial Intelligence (AI) tools in the creation of this article.
